# Pharmacogenetics May Prevent Psychotropic Adverse Events in Autism Spectrum Disorder: An Observational Pilot Study

**DOI:** 10.3390/ph16101496

**Published:** 2023-10-20

**Authors:** Laura de Miguel, Pura Ballester, Cecilia Egoavil, María Luisa Sánchez-Ocaña, Ana María García-Muñoz, Begoña Cerdá, Pilar Zafrilla, Enrique Ramos, Ana M. Peiró

**Affiliations:** 1 Pharmacogenetic Unit, Clinical Pharmacology Department, Alicante Institute for Health and Biomedical Research (ISABIAL), General University Hospital of Alicante, c/Pintor Baeza, 12, 03010 Alicante, Spain; 2 Clinical Pharmacology, Toxicology and Chemical Safety Unit, Institute of Bioengineering, Miguel Hernández University, Avda. de la Universidad s/n, 03202 Elche, Spain; 3 Faculty of Pharmacy and Nutrition, Campus de los Jerónimos, Universidad Católica San Antonio de Murcia (UCAM), Guadalupe, 30107 Murcia, Spain; 4 Clinical Pharmacology Unit, Dr. Balmis General University Hospital, 03010 Alicante, Spain

**Keywords:** autism spectrum disorder, pharmacogenetics, adverse events, polypharmacy, dopaminergic system

## Abstract

Introduction: Up to 73% of individuals with autism spectrum disorder (ASD) and intellectual disability (ID) currently have prescriptions for psychotropic drugs. This is explained by a higher prevalence of medical and psychiatric chronic comorbidities, which favors polypharmacy, increasing the probability of the appearance of adverse events (AEs). These could be a preventable cause of harm to patients with ASD and an unnecessary waste of healthcare resources. Objective: To study the impact of pharmacogenetic markers on the prevention of AE appearance in a population with ASD and ID. Methods: This is a cross-sectional, observational study (n = 118, 72 participants completed all information) in the ASD population. Sociodemographic and pharmacological data were gathered. The Udvalg for Kliniske Undersøgelser Scale (UKU Scale) was used to identify AEs related to the use of psychotropic medication. Polymorphisms of *DOP2*, *ABCB1*, and *COMT* were genotyped and correlated with the AE to find candidate genes. Furthermore, a review of all medications assessed in a clinical trial for adults with autism was performed to enrich the search for potential pharmacogenetic markers, keeping in mind the usual medications. Results: The majority of the study population were men (75%) with multiple comorbidities and polypharmacy, the most frequently prescribed drugs were antipsychotics (69%); 21% of the participants had four or more AEs related to psychotropic drugs. The most common were “Neurological” and” Psychiatric” (both 41%). Statistical analysis results suggested a significant correlation between the neurological symptoms and the *DOP2* genotype, given that they are not equally distributed among its allelic variants. The final review considered 19 manuscripts of medications for adults with ASD, and the confirmed genetic markers for those medications were consulted in databases. Conclusion: A possible correlation between neurologic AEs and polymorphisms of *DOP2* was observed; therefore, studying this gene could contribute to the safety of this population’s prescriptions. The following studies are underway to maximize statistical power and have a better representation of the population.

## 1. Introduction

Autism spectrum disorder (ASD) is a lifelong neurodevelopmental disorder that involves deficits in social interactions and repetitive/restricted behaviors [1]. The estimated global prevalence is 1–2%, varying widely among different countries and ethnicities. Numerous studies have reported an increasing tendency that is expected to keep growing in the coming years, positioning this pathology as a focal point of public health [2]. ID is defined as a deficit in adaptive functioning. Both cause impairment in different areas and are often diagnosed during the developmental period. ID and ASD co-occur in up to 30% of cases [3,4,5].

These individuals usually have prescriptions for psychotropic drugs in the context of symptomatic treatment for irritability or behavior disorders [6]. Those prescriptions in this population increase with age, and polypharmacy rates vary from 5 to 55% [4,6]. Both polypharmacy and comorbidity are very common [7], which elevates the appearance of drug–drug interactions and AEs, such as weight gain, motor disorders, hyperprolactinemia, or similar [8,9]. Usually, prescriptions imply a wide number of ongoing medications like antidepressants, anxiolytics, antipsychotics, or relapse control drugs, despite the little evidence of their efficacy in ASD [10,11]. Here, risperidone is the most commonly used drug for symptoms (irritability, aggression, and repetitive behaviors), being one of the two active ingredients with its use is approved for ASD core symptoms by regulatory drug agencies [12]. Methylphenidate, guanfacine, atomoxetine, clonidine, and naltrexone are normally used for Attention Deficit Hyperactivity Disorder in adults [13], as well as selective serotonin reuptake inhibitors, buprenorphine, alpha-adrenergic antagonists and tricyclic antidepressants, for the anxiety and depression disorders [14]. Given the complexity of managing multiple drugs, increasing adherence to treatments, and overall raising the quality of life of these patients, pharmacogenomics and pharmacogenetics are transpiring as a novel approach [15].

Given these complex prescription patterns, pharmacogenetics is an emerging field in ASD, as it intends to customize treatment according to the genetic profile of the patient, being an option for individualized medicine and having an effect on drug metabolism, efficacy, and safety [15]. Given approved medications for ASD, candidate genes should be related to the dopaminergic system. *DOP2,* which codes for the dopamine receptor D2, is involved in the action mechanism of the above-mentioned medications, e.g., risperidone [16]. Furthermore, *COMT* is an enzyme that participates in the metabolism of dopamine [17] together with the metabolism of several drugs, as well as posing a risk for drug–drug interactions [18]. On the other side, *ABCB1*, also known as the multidrug resistance protein 1, codes for P glycoprotein, which acts as a main transporter to the brain for these medications [19].

Briefly, the implementation of pharmacogenetic interventions could have the potential to significantly improve the clinical outcomes in severe comorbid ASD populations with drug treatment resistance and poor prognosis. Apart from that, we systematically gathered all medications studied under a clinical trial basis in adults with ASD and the pharmacogenetic markers. We proceed to study the impact of some pharmacogenetic markers on the appearance of AEs in the population with ASD and ID.

## 2. Results

### 2.1. Theoretical Review

**Figure 1:**
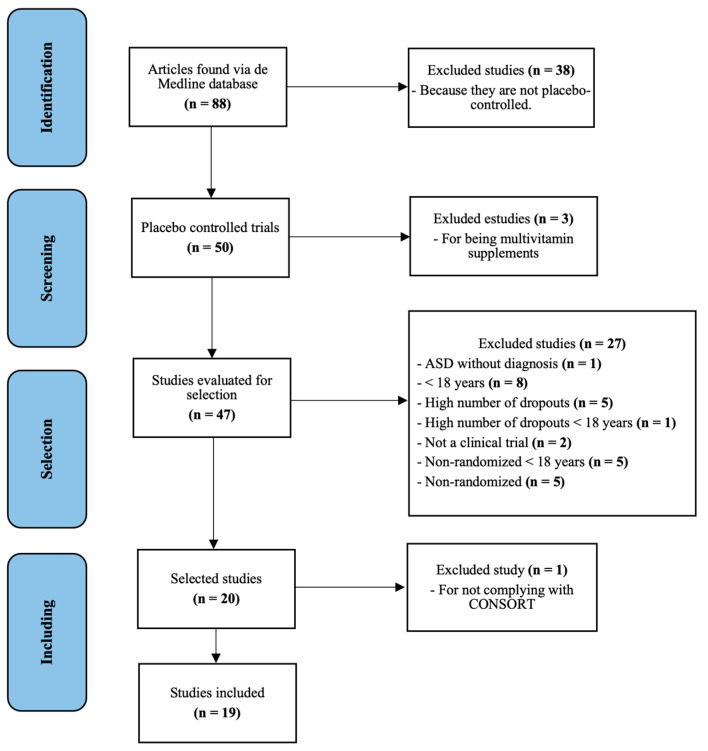
PRISMA flow diagram of all randomized clinical trials in adults with ASD.

The search performed generated 86 results; 38 were eliminated as they were not placebo-controlled. Of the remaining 48 articles, 3 studies were excluded because they involved a non-pharmacological intervention. The full text of 45 articles was evaluated, of which 27 were excluded because they did not meet the inclusion criteria mentioned above (see Figure 1 for detailed reasons for exclusion). Of the 18 articles resulting from this selection, 1 article was excluded because it did not meet the minimum quality criteria considering the items of the CONSORT guide. Finally, 19 articles met all the requirements for the study (see Figure 1).

The quality of the articles evaluated by CONSORT15 is shown in Table S1 of the Supplementary Materials. The compliance results of CONSORT items ranged from 52% to 84%. Here, one manuscript was excluded by meeting only 48% of the CONSORT specifications, and the lack of relevant information displayed reduced the reproducibility of the study.

### 2.2. Demographic and Pharmacological Outcomes

Patients’ demographics are presented in Table 1. Most of the study population were men, representing 75% of the total (54 patients). All patients have a diagnosis of ASD and ID according to Spanish social service records; further, a member of the research team confirmed this by clinical diagnosis. Among the cardiovascular risk factors (CVRFs), the most common was dyslipidemia (21%). None of the patients referred toxic habits (including smoking, alcohol consumption, and other drugs). Regarding their comorbidities, the most frequent was presenting five or more additional comorbidities, with a 29% representation over the total. Overall, the most prevalent comorbid diagnosis related to the cardiovascular system was dyslipidemia. A third of participants presented central nervous system comorbidities, such as anxiety or depression disorders, with epilepsy as the most prevalent condition at 20%. Urinary and digestive comorbid conditions were also common, at 13% and 16%, respectively, with conditions such as urinary incontinence or constipation, to name a few.

The most prescribed drugs were antipsychotics (69%), specifically risperidone (24%), the medication most prescribed among them. The most frequently used anticonvulsant was valproic acid (15%); the most frequently used antidepressant was fluvoxamine (8%); and finally, clonazepam stands out among the anxiolytics (12%).

### 2.3. Adverse Events

Data related to AEs were registered through the UKU scales; the most reiterated was the absence of AEs (29%), followed by the occurrence of four or more AEs (21%), as shown in Table 2.

The most usual AEs were the neurological AEs, especially epileptic seizures (41%), followed by psychiatric AEs, with the appearance of tension/restlessness commonly described in 48% of the responses. Finally, regarding the Autonomic AEs, constipation stands out, with a 55% share of the cases.

### 2.4. Pharmacogenetic Data

Most of the study population was heterozygous for the *DOP2* gene (43%) as well as for the *ABCB 1.2* gene (52%). On the other hand, most of the subjects were classified as wild-type for the *ABCB 1.1* gene (98%). As for the results for *COMT*, the most common was mutant, with 49% of the total; see Table 3. All polymorphisms presented participants in at least two of the three possible genotypes, and all genetic variants were at the Hardy–Weinberg Equilibrium. A comparison between allele frequencies of all pharmacogenetic variants included in the study and reference values (European population) can be found in Table S1 of the Supplementary Materials.

When correlating pharmacological data (number of simultaneous medications) with registered AEs, we found that individuals who were being administered between one and three medications simultaneously had a higher incidence of AEs. However, despite this observation, statistical analyses revealed no significant statistical differences directly related to these sociodemographic factors. We also established a correlation between individuals’ genotypes and the count of AEs registered using the UKU scale. The results of this analysis (*p*-values) are presented comprehensively in Table 4.

When contrasting genotype distribution for all the analyzed genes with the results obtained using the UKU scale for neurological, psychiatric, autonomic, or other AEs, no significant differences were found, except for *DOP2.* When correlated with neurological AEs, the relationship was statistically significant, meaning that neurological AEs were not equally distributed between the *DOP2* genotypes, which will require further analysis to confirm these differences found and which genotype has the highest probability of presenting a neurological AE.

All randomized clinical trials carried out in adults with ASD were reviewed for the present manuscript, and pharmacogenetic markers related to medication efficacy, safety, or metabolism are shown in Table 5.

**Figure 2:**
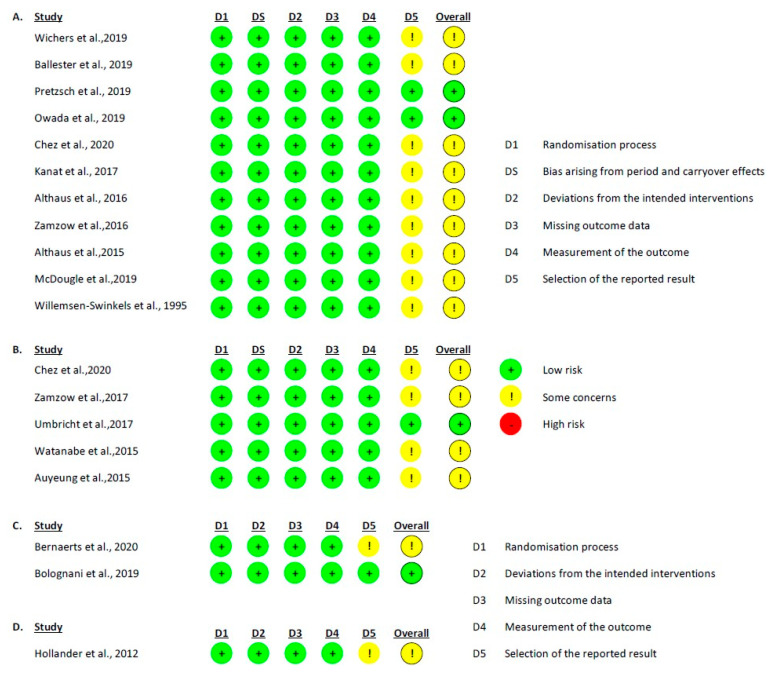
RoB 2.0 graph, showing the risk of bias analysis for the studies included in the review: (**A**) crossover *intention to treat* studies [21,22,23,24,26,28,31,32,33,36,38]; (**B**) crossover *per protocol* studies [26,29,30,34,35]; (**C**) parallel *intention to treat* studies [20,25]; and (**D**) parallel *per protocol* studies [37].

The risk of bias assessment for the randomized studies was performed using the RoB 2.0 tool. This analysis showed that the predominant risk of bias between studies was low for both parallel and crossover studies (Figure 2).

## 3. Discussion

Most of the ASD and ID population presented multiple comorbidities and several simultaneous ongoing medications, mainly antipsychotics. Nearly a third of the patients showed four or more AEs related to psychotropic drugs, where DOP2 allelic variants could influence the Neurologic AE appearance rate. A pharmacoeconomic study could be carried out from the perspective of the National Health System to assess the clinical translation of an anticipated dopaminergic genotyping in this vulnerable population.

We should consider that the literature concerning the general health conditions of adults with ASD is scarce. In this study, the mean age was 39 years old, higher than most published studies with a predominantly masculine population, in a similar way to prior data. ASD is more prevalent in males than females; however, recent research indicates that in females, camouflage of ASD symptoms is more prevalent than in males, potentially contributing to the differences in prevalence yet described [7,17,39]. This should be carefully analyzed through a sex and gender perspective analysis.

Though the study population lived in residential facilities, the exposure to substance abuse is lower, in line with what has been stated for ASD adults’ consumption of tobacco/alcohol/drugs; therefore, there is a lesser possibility of presenting a comorbid condition surrogated to this behavior. Croen et al. found a significant increase in the prevalence of notable chronic conditions in ASD subjects compared with controls, with comorbidity being the general rule. The number of comorbidities was consistent with our results; nonetheless, the order of importance amongst cardiovascular risk factors differed, dyslipidemia being the most common in comparison with other studies that positioned obesity and hypertension as the primary factors [7,39].

Regarding the pharmacological profile of our population, we found consistent results with other studies, where the most prescribed medications were antipsychotics, risperidone standing out among them. Nevertheless, those studies reported the use of three or more drugs simultaneously in approximately 10% of the patients, whereas in our project, the number of patients in this situation rose up to 46% [2,40,41].

The genotype frequencies of *ABCB1* were similar to previous studies located in Spain [19]. The T allele of *ABCB1*.2 has been associated with a lower expression of P-gp (present in the blood–brain barrier), resulting in higher concentrations of medications that constitute a substrate to this protein, such as several antidepressants and antipsychotics. These increased drug levels surpassing the recommended range in plasma, and especially in the brain, could be associated with more AEs. However, other studies have failed to establish a clear association. Most participants of the study presented a genotype AG for this variant; authors have linked this genotype with reduced social and clinical needs in participants treated with antipsychotics [42]. In patients with a mutant genotype, more insomnia and fatigue have been reported by Lin et al., though these results have not been replicated [43]. The wild-type and heterozygous variants of ABCB1.1 have been associated with higher antidepressant plasma levels, though they did not reach a significant result in the appearance of AEs or treatment responses [44,45]. Also, for this genotype, authors have described an increased likelihood of drug resistance when treated with antiepileptics [46] and increased AE rates related to antipsychotics [47]; therefore, participants may require a lower dose to avoid toxicity. These results match our own, as we found no significant associations between *ABCB1* genotypes and AEs, taking into consideration that we found no studies with a similar population whose aim was to correlate these two variables.

When analyzing the *COMT* gene, we found that 49% of our population was mutant (AA), as opposed to previous studies, at the expense of heterozygous subjects [17,48]. *COMT* has been associated with elevated proportions of tension/restlessness, anxiety, and depression in individuals not diagnosed with ASD. However, this was not supported when performing studies on participants with ASD taking four or more simultaneous medications, which is consistent with our result, having found no significant correlation between psychiatric symptoms and COMT polymorphisms [49]. Esmaiel et al. reported the mutant variant of *COMT* being associated with increased levels of dopamine and abnormalities in EEG, suggesting a significantly elevated prevalence of epilepsy as well as a decreased seizure threshold which could result in more motor and neurological AEs. Furthermore, in line with our results, it has been described that those who are AA and treated with antipsychotics may experience more frequent metabolic syndromes than those who have a GG genotype [50].

Finally, with respect to *DOP2*, there are very few studies despite it being a crucial candidate gene [51]. It has been associated with seizures, motor disorders, and pathophysiology of ASD, but to this date, there are no studies aiming to correlate AEs with allelic variants, except two studies with inconclusive results on hyperprolactinemia when treated with risperidone [4,16,51]. AG genotype has been linked to weight gain when treated with antipsychotics, compared to the other genotypes [52]. To the present day, we have not found studies correlating autonomic AEs with a patient´s *DOP2* genotype [40].

We found a low number of nineteen clinical trials with pharmacological therapy in adults with ASD that met the inclusion criteria. The methodological quality of these trials is high, which is reflected in the value of the CONSORT guide. Most of these existing pharmacological clinical trials on ASD have been conducted in the last five years, so they are in the early stages of research. They usually include a low number of participants together with multiple interventions or designs, which makes it difficult to demonstrate their efficacy by comparison at a general level. Therefore, it would be necessary to carry out more studies with a larger sample size plus long-term treatment. These clinical trials were on medications that will benefit from the genetic analysis proposed in this study.

The main limitation was the sample size of the statistically analyzed group, which translated into lower statistical power. The difficulties of separating symptoms from AEs due to underlying illnesses potentially caused by medications were a challenge as well. The research team identifying some of the items of the UKU Scale that were finally categorized as “not easily observable” by the healthcare professionals represented a challenge as well. Finally, another possible limitation of our study is that our theoretical review was based only on the PubMed of the MEDLINE and Scopus databases. Future research could benefit from exploring a larger number of databases to potentially access more articles.

Concerning the application of a pharmacogenetic testing approach to daily clinical practice, several studies have assessed the acceptability and feasibility of this practice [18,53,54]. The identification of genetic variants of enzymes, receptors, and transporters may provide useful information for dosage and duration of treatment, as well as predictions on therapy outcomes and side effects. Currently, specific factors or AEs are being targeted to estimate the relevance of certain genes, though, in the future, we can expect the development of panels addressing general characteristics, faster screening methods, and protocols for applying these findings [54,55,56].

The study of pharmacogenetics in ASD and ID populations is booming, emerging as an innovative perspective for personalized medicine. A pharmacoeconomic study could be carried out from the perspective of the National Health System to assess the clinical translation of an anticipated dopaminergic genotyping. Furthermore, as recent research has stated some specific deficiencies compared to the control group in the cognitive profile, and behavioral and emotional problems have been described in ASD, adding the genotype perspective would be an interesting point in evaluation [57].

## 4. Materials and Methods

### 4.1. Study Design and Ethics

An observational ambispective study was conducted for 36 months from January 2015 to January 2018, consisting of 36 months of retrospective revision of electronic health records and 12 months of prospective follow up carried out from November 2019 to November 2020. All adults with ASD were inhabitants of a residential facility, Infanta Leonor, San Rafael, APNAV, or EDUCATEA, and attended the Alicante Department of Health–General Hospital (Alicante, Spain).

The Ethics Committee board of Alicante Department of Health–General Hospital and UCAM University approved the protocol and all procedures of this study (Ethic Committee code 2016/02 and CE022211, respectively). All patients and/or legal representatives signed the consent form. Further details of the local pharmacovigilance system created have been previously reported in another work [2].

### 4.2. Participants

The participants included came from residential facilities located in Santa Faz (Alicante, Spain), following inclusion criteria: 18 years old or above; having a diagnosis of ASD as established by the DSM-5; having a diagnosis of an intellectual disability (IQ < 70 points); and the patient/legal representative has received, understood, and signed the consent form. Also, the following exclusion criteria were applied: patients with a medical condition or development of their pathology that made it difficult to participate (e.g., aggressive behavior, discomfort symptoms such as pain, or severe ASD core symptoms). All participants could withdraw from the study at any time.

### 4.3. Procedure

Six pharmacists and one clinical pharmacologist comprised the research team. They contacted the ASD centers and held meetings with the legal representatives/family/social services to inform them about the objectives of the present study and to give the appropriate information that would guarantee signed informed consent. Once it was obtained, in collaboration with the healthcare team, the electronic or paper medical records were reviewed to collect the study variables. Participants, families, and health providers were subsequently informed about the results of the study in different individual interviews and regular center meetings.

### 4.4. Data Collection

#### 4.4.1. Sociodemographic and Pharmacological Data

Demographic data, including age, sex, cardiovascular risk factors, medical comorbidities, and toxic habits, were obtained during routine clinical visits. Information regarding participants’ ongoing medications was collected from electronic health records between November 2019 and April 2020. At that moment, the UKU Scale was filled for some participants [58]. The UKU scale is utilized to systematically assess and rate the side effects of psychotropic medications. These items are grouped into categories like psychiatric, neurological, autonomic, and other side effects. Each item is rated based on its severity and its possible association with the medication. Drugs were classified according to their main indication available in each technical data sheet. For this study, we considered antipsychotics, anxiolytics, antidepressants, and anticonvulsants.

#### 4.4.2. Adverse Events

The difficulties in communication and the introspection characteristics of these patients should be taken into consideration. Tveter, Bakken et al. [59] proposed an adjustment of this scale that classified the symptoms regarding observability and difficulty to score for the nurses and experts. Thus, to evaluate the presence of AEs, an adaptation to the UKU Scale for patients with intellectual disabilities was used [58]. This scale constitutes a measurement of the secondary events, both physical and psychic, that the intake of psychotropic drugs can produce. There are no cut-off points to this scale; the higher the mark, the more severe the AEs are. The modified scale focused on “The single symptom rating scale”, covering four different areas: psychic, neurological, autonomic, and other side effects [58]. Each of the items was scored as follows: 0: not present, 1: very occasionally present, 2: present in a mild degree, 3: present twice a week, 4: present 4 times a week, 5: present every day, and 99: not appropriate or relevant.

#### 4.4.3. Pharmacogenetics Markers

The analysis of the polymorphisms of dopaminergic receptor genes or enzymes in charge of medication metabolism or medication transport was performed, including *DOP2*, *COMT*, ABCB 1.1, and *ABCB1*.2 from blood samples; see variant information in Table 6. The candidate gene *DOP2* was elected according to previous studies that pointed to the affinity of risperidone [60] and aripiprazole [61]. The study of *COMT* was considered according not only to the known mechanism of action of this enzyme, as previously mentioned, but also due to the evidence described for ADHD [62,63]. Finally, the *ABCB* gene was studied as it has been described as a relevant action of this gene for patients treated with risperidone [64]. The Kit for DNA extraction of blood samples was used, and the samples were analyzed through TaqMan1, using a real-time PCR system (Thermo Fisher Scientific Inc., Waltham, MA, USA).

### 4.5. Theoretical Review

The theoretical review was carried out using the search engine Pubmed of the MEDLINE database and Scopus to ensure the retrieval of the maximum number of published manuscripts. The search was first conducted in January 2021, and then a second one in June 2022 to update the results with the following equation: (“autism spectrum disorder/drug therapy” [MeSH Terms]) AND ((clinical trial [Filter]) AND (humans [Filter]) AND (alladult [Filter])).

The procedures to select manuscripts included in this work were all clinical trials performed for (a) adults (age > 17 years old) (b) with ASD demonstrated by criteria published in the Diagnostic and Statistical Manual of Mental Disorders (DSM-51 and DSM-IV14) and confirmed using the Autism Diagnostic Interview Revised (ADI-R12) and/or Autism Diagnostic Observational Schedule (ADOS-2), (c) randomized with a control group (either placebo or standard treatment) against the intervention drug, (d) with the efficacy of their pharmacological response producing a change at the neurobiological and behavioral level in ASD evaluated, and (e) achieving a threshold of a 50% score in the requirements of the Consolidated Standards of Reporting Trials (CONSORT15). All works not compliant with these criteria were considered excluded manuscripts in the present work.

The evaluation process of the articles retrieved through searches began by examining their titles and keywords. Following that, abstracts of selected articles were screened. The final step involved reading the full text of potential manuscripts and assessing them using the CONSORT guidelines.

To assess the potential risk of bias in the selected studies, we used the Cochrane tool (RoB 2.0, parallel or crossover, depending on the type of study) [65] for randomized clinical trials.

### 4.6. Statistical Analysis

An optimal sample size was not determined for this study. All study participants were patients at the hospital where the research was conducted, and the size of the sample was in line with previous studies pursuing similar goals. The symptomatology scale (psychiatric, neurological, autonomic, or similar) was categorized as a dichotomous variable (presents symptoms yes/no) for each symptomatic group. Each gene’s allele was categorized as a qualitative variable of 3 categories (the heterozygous variable and two homozygous variables for the given allele). This gives us a resulting statistical analysis when combining each dichotomous variable of the symptomatic groups with qualitative variables (3 categories) of allele variants.

To prove if there are significant differences in a binary variable between more than 3 independent groups, it is common to use the Chi-Square test; however, in our model, there are only n < 30 subjects obtaining expected values lower than 5; therefore, the Chi-Square test is not viable (statistical significance could not be achieved complying with both conditions). Given the above, the Freeman Halton extension of Fisher’s Exact Test with Yates correction was used to calculate the probability (two-tailed) to obtain a value distribution in a contingency table, given the observations on each cell.

This results in a series of statistical analyses (4 symptomatic groups yes/no) × 4 genes (*DOP2*, *COMT*, *ABCB1.1*, and *ABCB1.2*) = 16 hypothesis contrasts. This analysis was performed only to explore relevant relationships between genes and AEs. Hardy–Weinberg Equilibrium was assessed using the equation and considering the final Chi-Square value with a degree of freedom. All analyses were carried out in the software R studio 3.3.0; significant differences were considered when *p*-value was <0.05.

## 5. Conclusions

Our data evidenced that most of the study population were men with multiple comorbidities, amongst which the most prevalent was dyslipidemia, being under two or three drugs co-prescription, mostly antipsychotics. These findings are supported by previous studies with similar results and should be analyzed through a sex/gender perspective. The highest percentage of a third of the subjects had no AEs, but nearly a quarter presented four or more, mostly related to neurological and psychiatric areas, specifically epileptic seizures and restlessness. This different safety profile should be analyzed more deeply through personalized medicine. Statistical analysis results suggested a significant correlation between the neurological symptoms and the *DOP2* genotype, given that the alleles were not equally distributed. Follow up studies are underway to maximize statistical power with a bigger sample size, which we expect will corroborate these results or suggest new hypotheses. Furthermore, they would contribute to a differential ASD prescription and tolerability. Considering these potential interactions plus subsequent monitoring could help us to understand the interindividual variability in autistic real-world subjects.

## Figures and Tables

**Table 1:** Demographic and pharmacological data.

Variables (n = 72 Participants)		
Sex (n, %)	Male	54 (75%)
	Female	18 (25%)
Age (mean ± standard dev)		39.1 ± 13.2
Cardiovascular risk factors (n, %)	Dyslipidemia	15 (21%)
	Obesity	14 (19%)
	Diabetes	4 (5%)
	Hypertension	4 (5%)
Comorbidities (n, %)	Without	4 (5%)
	1	8 (11%)
	2	9 (12%)
	3	16 (22%)
	4	14 (19%)
	5 or more	21 (29%)
Drug group (n, %)	Antipsychotics	81 (69%)
	Antidepressant	19 (14%)
	Anticonvulsants	41 (31%)
	Anxiolytics	19 (9%)
	Without	15 (13%)
Number of simultaneous medications (n, %)	2	27 (23%)
	3	25 (21%)
	4	13 (11%)
	5 or more	16 (14%)

**Table 2:** Number of adverse events (AEs, n = 34) from the UKU scale results.

Number of AEs	0	10 (29%)
	1	6 (18%)
	2	6 (18%)
	3	5 (15%)
	>4	7 (21%)
Number of AEs by AE group	Psychiatric	14 (41%)
	Neurological	14 (41%)
	Autonomic	12 (35%)
	Other	12 (35%)

**Table 3:** Genotypic and allelic frequencies of all pharmacogenetic variants included in the study.

Polymorphisms n = 100		n	% Study
*DOP2* rs6277	WT(GG)	37	40%
	MUT (AA)	16	17%
	HTZ (AG)	39	43%
	W/O data	8	NA
Alleles	G	113	61%
	A	71	39%
*COMT* rs4680	WT(GG)	21	41%
	MUT (AA)	25	49%
	HTZ (GA)	5	10%
	W/O data	49	NA
Alleles	G	47	46%
	A	55	54%
*ABCB1.1* rs2032582	WT(AA)	79	98%
	MUT(CC)	0	0%
	HTZ(AC)	2	2%
	W/O data	19	NA
Alleles	A	160	99%
	C	2	1%
*ABCB1.2* rs1045642	WT(AA)	20	24%
	MUT(GG)	20	24%
	HTZ(AG)	42	52%
	W/O data	18	18%
Alleles	A	82	50%
	G	82	50%

**Table 4:** (A) *p*-values obtained when correlating genotype and AEs and (B) expanding *DOP2* information.

(A)	*DOP2*	*COMT*	*ABCB1.1*	*ABCB1.2*
	*p*-value			
Psychiatric AE	1	0.3	1	0.4
Neurological AE	0.01	0.06	1	0.8
Autonomic AE	0.3	0.3	1	1
Other AE	0.8	0.3	1	0.8
**(B) Alleles of *DOP2***				
	GA	AA	GG	
	*p*-value			
Neurological AE	1.00	0.02	0.04	

AE: adverse event; *DOP*: dopamine receptor gene; *COMT*: catechol o-methyl transferase gene; GA: guanine–alanine genotype; AA: alanine–alanine genotype; GG: guanine–guanine genotype.

**Table 5:** All clinical trials of medication performed in adults with ASD together with pharmacogenetic markers related to efficacy, safety, or medication metabolism of the studies included in the systematic review.

Author, Year	Medication (Daily Dose)	Participants (N; % Male; Age ± SD)	Method (Design, Duration Months)	Diagnosis Tool	Results	Pharmacogenetic Markers
Bernaerts et al., 2020 [20]	Oxytocin (24 IU/day) vs. placebo	N = 40 (100%M) 27 ± 9	Parallel, double-blind, randomized, and placebo-controlled, 4 weeks	DSM-IV-TR and ADOS	Decreased social avoidance, repetitive behaviors, and mood improvements.	NA
Wichers et al., 2019 [21]	Citalopram (20 mg/day) vs. placebo	N = 38 (100%M, 19 controls and 19 ASD); 27 ± 9 and 30 ± 11	Crossover, double-blind, randomized, and placebo-controlled	ADI-R and ADOS	Activity normalization of the inferior frontal cortex, and functional differences in the brain were abolished.	*CYP2C19, CYP2D6*
Ballester et al., 2019 [22]	Agomelatine (25 mg/day) vs. placebo	N = 23 (83%M) 35 ± 12	Crossover, triple-blind, randomized, and placebo-controlled, 6 months	DSM-5-TR criteria	Improves sleep quality, and total sleep time increased by an average of 83 min.	*ABCB1*
Pretzsch et al., 2019 [23]	CBD (600 mg/day) vs. placebo	N = 34 (100%M; 17 controls and 17 ASD) 28 ± 7 and 31 ± 10	Crossover, double-blind, randomized, and placebo-controlled	ADOS and ADI-R	Changes in low-frequency brain fluctuations in the cerebellar vermis and in the right fusiform gyrus in individuals with ASD.	*AOC1, ABCC5, SLC15A1*
Owada et al., 2019 [24]	Oxytocin (24 IU/every 12 h) vs. placebo	N = 18 (100%M, N = 9 and N = 9) 35 ± 7 and 29 ± 6–N = 103 (100%M, N = 51 and N = 52) 27 ± 7 and 26 ± 7	Crossover, double-blind, randomized, placebo-controlled, multicenter, parallel, double-blind, and placebo-controlled, 6 weeks	DSM-IV-TR	Decreases the likelihood of neutral facial expression during social interaction.	NA
Bolognani et al., 2019 [25]	Balovaptan (1.5, 4, or 10 mg/day) vs. placebo	N = 223.(100%M) 24 ± 2	Phase 2, parallel-group, multicenter, double-blind, randomized, and placebo-controlled. Stage 1: 1.5 mg balovaptan vs. placebo (2:1), Stage 2: 4 mg vs. placebo (2:1), Stage 3: 10 mg vs. placebo (2:1), and Stage 4: 1.5 or 10 mg vs. placebo (1:1:1), 12 weeks each	DSM-5-TR and ADOS-2	Dose-dependent improvement of balovaptan compared to placebo was observed in standard scores in the socialization and communication domains.	NA
Chez et al., 2018 [26]	Dextromethorphan (20 mg)/quinidine (10 mg) vs. placebo every 24 h the first week and every 12 h the second week	N = 15 (78%M) 39 ± 21	Crossover, double-blind, randomized, and placebo-controlled, 24 weeks	DSM-IV-TR and ADOS scale	Decreased irritability and aggression; associated with significant behavioral improvements in individuals with autism.	*CYP2D6*
Quintana et al., 2017 [27]	Oxytocin (8 IU or 24 IU/day) vs. placebo	N = 17 (100%M) 27 ± 8	Crossover, double-blind, randomized, and placebo-controlled	DSM-IV	Breath-powered administration increases bioavailability and improves perception of emotion sensitivity significantly at an 8 IU dose. There were no significant changes in facial expression grading speed.	NA
Kanat et al., 2017 [28]	Oxytocin (24 IU/day) vs. Placebo	N = 59 (100%M, N = 29 ASD and N = 30 controls) 38 ± 11 and 32 ± 12	Crossover, double-blind, randomized (in blocks of 10), and placebo-controlled	DSM-IV	Oxytocin increased attention to faces in the face in ASD individuals. The effects of oxytocin were greater in ASD participants with high levels of social anxiety, who were characterized by avoidance of attention to faces under placebo.	NA
Zamzow et al., 2017 [29]	Propranolol (40 mg) vs. placebo	N = 20 (95%M) 21 ± 5	Crossover, double-blind, randomized, and placebo-controlled	ADI-R and DSM-IV	Participants resolved more easily in the propranolol arm. No effects on anxiety were found.	*CYP2D6*
Umbricht et al., 2017 [30]	Vasopressin receptor antagonist RG7713 (20 mg intravenous) vs. placebo	N = 19 (100%M) 23 ± 5	Multicenter, crossover, double-blind, randomized, and placebo-controlled	ADOS and DSM-IV	Statistically significant effects were limited to improved attention to relevant biological information and social cognition.	NA
Althaus et al., 2016 [31]	Oxytocin (24 IU/day intranasal) vs. placebo	N = 61 (100%M, N = 31 ASD and N = 30 controls) 23 ± 4	Crossover, double-blind, randomized, and placebo-controlled	(DSM)-IV and ADOS	There were no significant differences between oxytocin plasma levels and improvements in behavior or anxiety.	NA
Zamzow et al., 2016 [32]	Propranolol (40 mg) vs. placebo	N = 20 (95%M) 21 ± 3	Crossover, double-blind, randomized, and placebo-controlled	ADI-R and DSM-IV	Propranolol improved reciprocity and non-verbal communication. However, no differences were found in anxiety.	*CYP2D6*
Althaus et al., 2015 [33]	Oxytocin (24 UI/day) vs. placebo	N = 62 (100%M 30 control and 32 ASD) 23 ± 3 and 23 ± 5	Crossover, double-blind, randomized, and placebo-controlled	DSM-IV, ADOS, SRS-A, and AQ	No significant differences were found between groups, either social response or anxiety.	NA
Watanabe et al., 2015 [34]	Oxytocin (48 UI/day) vs. placebo	N = 20 (100%M 10 control and 10 ASD) 36 ± 19 and 36 ± 19	Crossover, double-blind, randomized, and placebo-controlled (duration: 6 weeks)	DSM-IV and ADOS-2	Improvement in social reciprocity.	NA
Auyeung et al., 2015 [35]	Oxytocin (24 UI/day) vs. placebo	N = 74 (100%M; 37 control and 37 ASD) 34 ± 9	Intraindividual, Crossover, double-blind, randomized, and placebo-controlled (duration: 2 weeks)	DSM-IV	Oxytocin significantly increases participants’ social eye looking in both groups.	NA
McDougle et al., 1996 [36]	Fluvoxamine (50–300 mg/day) vs. placebo	N = 30 (90%M) 30 ± 8	Crossover, double-blind, randomized, and placebo-controlled (duration: 12 weeks)	DSM-III-R, IQ with WASI, and overall status with CGI	A reduction in repetitive and aggressive behaviors was measured.	*CYP2C19, CYP2D6*
Hollander et al., 2012 [37]	Fluoxetine (10 mg–20 mg–80 mg/day) vs. placebo	N = 37 (69%M) 30 ± 3	Double-blinded and placebo-controlled trial (duration: 12 weeks)	CGI, Yale-Brown Obsessive Compulsive Scale, and DSM-IV	This drug was found to improve repetitive b behaviors and overall core symptoms with large effect sizes.	*CYP2D6*
Willemsen-Swinkels et al., 1995 [38]	Naltrexone (50–150 mg/day) vs. placebo	N= 33 (81%M) 29 ± 6	Crossover, double-blind, randomized, and placebo-controlled (duration: 4 weeks)	DSM-III-R	Increase in stereotyped behavior, no positive effects, and side effects that prompted losses of follow up.	* COMT * , *OPRM1*

ADI-R—Autism Diagnostic Interview Revised; ADOS—the Autism Diagnostic Observation Schedule; AQ—Autism Quotient; ASD—autism spectrum disorder; DSM-IV-TR—Diagnostic and Statistical Manual of Mental Disorders IV Edition; DSM-5-TR—Diagnostic and Statistical Manual of Mental Disorders 5th Edition; IQ—Intelligence Quotient; M—male; N—sample size; NA—not available; and OXT—oxytocin.

**Table 6:** Polymorphisms assessed in this study information.

Gene Name	rs ID	Reference Allele	Alternate Allele	MAF
*DOP2-DRD2*	rs6277	G	A	G = 0.4471 A = 0.5529 *
*COMT*	rs4680	G	A	G = 0.491334 A = 0.508666 *
*ABCB1.1*	rs2032582	A	C/T	A = 0.446826 C = 0.552030 T = 0.001144 *
*ABCB1.2*	rs1045642	A	G	A = 0.519742 G = 0.480258 *

* Allele frequencies were obtained from NCBI SNP database; all displayed for the European population, considered the reference group for study participants.

## Data Availability

Data is contained within the article.

## Supplementary Materials

The following supporting information can be downloaded at: https://www.mdpi.com/article/10.3390/ph16101496/s1, Table S1: Manuscript quality analyzed using the 25 items of the CONSORT guidelines.
