# Supplementary material for: Pharmacogenetics May Prevent Psychotropic Adverse Events in Autism Spectrum Disorder: An Observational Pilot Study
Source: Pharmaceuticals (Basel). 2023 Oct 20;16(10):1496. doi: 10.3390/ph16101496 (PMC10610471; doi:10.3390/ph16101496)
Supplement: Supplementary file 1 [file pharmaceuticals-16-01496-s001.zip › pharmaceuticals-2603403-supplementary.pdf]

Table S1: Manuscript quality analyzed using the 25 items of the CONSORT guidelines. Scoring: "0", "1", "0.5" or "X" on the irrelevant item.

**CONSORT GUIDELINES ANALYSIS**																											
**Item order**	**1**	**2**	**3**	**4**	**5**	**6**	**7**	**8**	**9**	**10**	**11**	**12**	**13**	**14**	**15**	**16**	**17**	**18**	**19**	**20**	**21**	**22**	**23**	**24**	**25**	**TOTAL**	**%**
Bernaerts et al., 2020 [28]	1	1	1	1	1	1	0	0.5	0	0	1	0.5	0.5	0.5	1	1	1	1	x	1	1	1	1	1	1	**19**	**79**
Wichers et al.,2019 [29]	0	1	1	1	1	1	0.5	0.5	1	0	1	1	0.5	0	0	1	1	1	1	1	1	1	0	1	1	**18,5**	**74**
Ballester et al., 2019 [30]	0.5	1	0.5	1	1	1	0.5	1	0	0	1	1	1	0.5	1	1	1	x	x	1	1	1	1	1	1	**19**	**83**
Pretzsch et al., 2019 [31]	0.5	1	1	1	1	1	0	0.5	0	0	1	1	0.5	0	0	1	1	1	x	1	1	1	1	1	1	**17,5**	**73**
Owada et al., 2019 [32]	0	1	1	1	1	1	0.5	0.5	0	0	1	0.5	0.5	0.5	1	1	1	1	x	1	1	1	1	1	1	**19,5**	**81**
Bolognani et al., 2019 [33]	0	1	1	1	1	1	1	0	0	0	1	0	1	0.5	1	1	1	1	1	1	1	1	1	1	0	**18,5**	**74**
Chez et al., 2018 [34]	0.5	1	0.5	0.5	1	1	0.5	0	0	0	1	0.5	1	0	0	1	1	0	1	1	1	1	0	0	0	**13.5**	**54**
Quintana et al.,2017 [35]	0.5	1	0.5	1	1	1	0.5	1	1	1	1	1	1	0.5	0	1	1	1	1	1	1	1	1	1	0	**21**	**84**
Kanat et al.,2017 [36]	0	1	0.5	1	1	1	0.5	1	1	1	1	1	0	0	1	1	1	1	0	1	0	1	1	0	0	**17**	**68**
Zamzow et al., 2017 [37]	0	1	0.5	1	1	1	0	0.5	0	0	0.5	1	1	0	1	1	1	x	0	1	0	0	0	0	1	**12,5**	**52**
Umbricht et al., 2017 [38]	0.5	1	0.5	1	1	1	0.5	0	0	0	0.5	0.5	1	0	1	1	1	1	1	1	1	1	1	0	1	**17.5**	**70**
Althaus et al.,2016 [39]	0.5	1	0.5	1	1	1	0	0	0	0	1	1	0.5	0	1	1	1	1	x	1	1	1	0	0	1	**15.5**	**64**
Zamzow et al., 2016 [40]	0.5	1	0.5	1	1	1	0	0	0	0	0.5	1	0.5	0	1	1	1	1	0	1	1	1	0	0	1	**15**	**60**
Althaus et al., 2015 [41]	0.5	1	0.5	1	1	1	0.5	0	0	0	0.5	1	1	0.5	1	1	1	1	0	1	0	1	1	1	0	**16.5**	**66**
Watanabe et al., 2015 [42]	0	1	0.5	1	1	1	1	1	0	1	1	0.5	1	0	1	1	1	1	1	1	1	1	1	1	1	**21**	**84**
Auyeung et al., 2015 [43]	0	1	0.5	1	1	1	0.5	0.5	0	0	0.5	0.5	1	0	1	1	1	1	1	1	1	1	0	1	0	**16.5**	**66**
McDougle et al., 1996 [44]	0.5	0.5	0.5	0.5	1	1	0	0	0	0	0.5	0.5	0.5	0.5	1	1	1	0	1	0	1	1	0	0	0	**12**	**48**
Hollander et al., 2012 [45]	1	1	1	0.5	1	1	0.5	0	0	0	0.5	0.5	1	0,5	1	1	1	0.5	1	1	1	1	0	0.5	0	**16.5**	**66**
Willemsen-Swinkels et al., 1995 [46]	0.5	1	1	0.5	1	1	0.5	0	0	0	1	0.5	1	0.5	1	1	1	1	1	1	1	1	0	0	1	**17.5**	**70**
